# A diagnostic dilemma: acute abdomen presenting as segmental arterial mediolysis masked by a ruptured hepatocellular carcinoma

**DOI:** 10.1093/gastro/gov030

**Published:** 2015-07-10

**Authors:** Chen-Yi Liao, Wu-Hsien Kuo, En-Hua Huang, An-Tie Hsieh, Ching-Chang Le, Chi-Chang Tsai, Chao-Wen Hsueh

**Affiliations:** Department of Medicine, Kaohsiung Armed Forces General Hospital, Kaohsiung, Taiwan

**Keywords:** segmental arterial mediolysis, hepatocellular carcinoma

## Abstract

A 65-year-old male was brought to our hospital with right upper abdominal fullness sensation and recent body weight loss of about 3 kg. The patient had developed episodes of melena following progressive abdominal muscular guarding and drop of haemoglobin level to 6.3 g/dL. An abdominal computed tomography scan disclosed a ruptured hepatocellular cell carcinoma. A segmental arterial mediolysis was found on the superior mesenteric artery in the process of repairing the ruptured right hepatic artery with the assistance of angiography. Transarterial embolization was carried out and permanent haemostasis was achieved.

## Introduction

Segmental arterial mediolysis (SAM) is an uncommon non-inflammatory and non-atherosclerotic vascular disease first described by Slavin in 1976, which is characterized by dissecting aneurysms, haematoma, occlusion and haemorrhage following vacuolization, and lysis of the outer media of the arterial wall (arterial media). It occasionally occurs in the middle-aged and elderly, but rarely in young patients [1]. A 26–50% mortality rate has been reported in the acute phase [2, 3]. SAM often involves more than one small- to medium-sized muscular abdominal and visceral artery, such as branches of the celiac artery and superior mesenteric artery, exhibiting a skip pattern. Arterial involvement is largely abdominal or cranial, with splenic arterial involvement being the most prevalent [4–5]. Others include the retroperitoneal, pulmonary, and coronary arteries [6–8]. SAM is a mimic, co-existing with several entities of vascular disease and rarely accompanied by malignancy. We present herein a case of initial diagnosis of ruptured hepatic cellular carcinoma and subsequent angiography leading to the incidental finding of a segmental arterial mediolysis on the superior mesenteric artery.

## Case presentation

**Figure 1.**
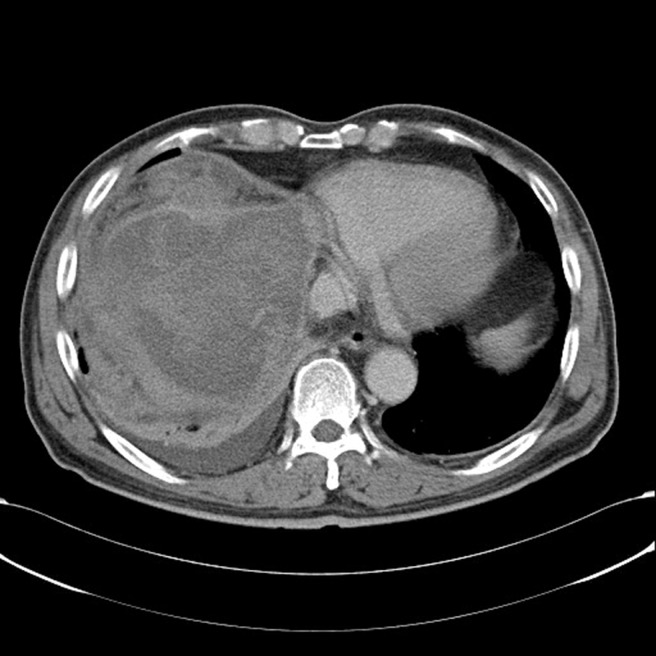
Abdominal computed tomography showed a huge, ruptured hepatocellular carcinoma with haemoperitoneum.

**Figure 2.**
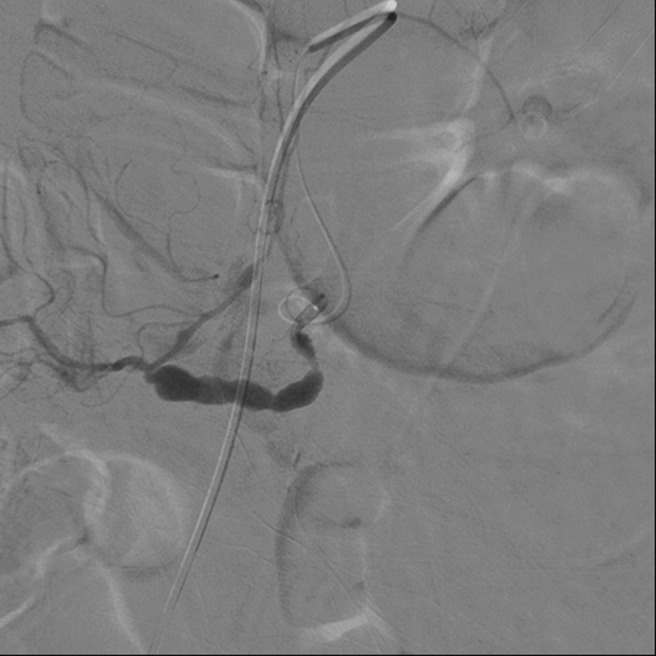
Angiography revealed an aneurysm with ‘string-of-beads’ appearance on the superior mesenteric artery.

**Figure 3.**
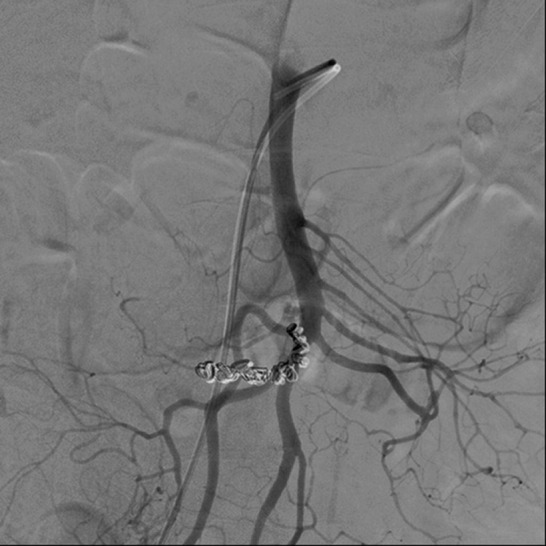
Second-time angiography showed successful vascular embolization of the lesion site with an intravascular coil.

A 65-year-old male, who had previously been in good health, presented to the emergency department with a 2-month history of weight loss of about 3 kg. He reported poor appetite and a distended sensation over the right upper quadrant region of the abdomen. General vital signs revealed a body temperature of 37°C, a rapid heart rate of 109 beats per minute, and a blood pressure of 129/90 mmHg. Physical examination revealed a distended right upper epigastric region with a palpable mass lesion. No muscular guarding or tenderness was observed initially. Laboratory examination results are shown in Table 1. During hospitalization, the patient developed episodes of melena following progressive abdominal muscular guarding diffuse tenderness and a reduction in haemoglobin from an initial 9.7 g/dL to 6.3 g/dL. Emergency upper gastrointestinal endoscopy produced unremarkable findings; due to the previously-mentioned palpable mass and a sudden, unexplained drop in haemoglobin level, the patient subsequently underwent abdominal computed tomography (CT), which revealed a huge, ruptured hepatocellular carcinoma about 13.5 cm across, occupying the right lobe of the liver, with observable haemoperitoneum (Figure 1). The patient received prompt blood transfusion with 4U packed red blood cells and 4U fresh frozen plasma, and was referred to the radiography department for further possible vascular intervention and haemostasis. Emergency embolization of the right hepatic artery with microcoils achieved complete arrest of extravasation. Further angiography incidentally revealed an aneurysm resembling bead strings over the superior mesenteric artery, which is compatible with the appearance of SAM (Figure 2). The patient had an uneventful hospital course after the segmental arterial mediolysis over the superior mesenteric artery had been treated by selective transcatheter arterial coil embolization (Figure 3).

**Table 1.** Laboratory parameters

Laboratory parameters	Admission	Normal range
Blood		
White blood cell, x 10^3^/μL	7.5	4.0–11.0
Haemoglobin, g/dL	9.7	13.5–17.5
Platelet, x10^3^/μL	292	150–400
Total bilirubin, mg/dL	5.92	0.3–1.0
Alanine aminotransferase, U/L	99	7–52
Aspartate aminotransferase, U/L	140	13–39
Creatinine, mg/dL	1.5	0.7–1.3
C-reactive protein, mg/dL	14.90	<1.0
Albumin, g/dL	2.9	3.5–5.7
alpha-Fetoprotein, ng/mL	635.12	1.09–8.04
CA-199, U/mL	39.76	0–37
Urinalysis		
Glucose	(−)	(−)
Protein	1+	(−)
Urobilirogen	2+	(−)
Bilirubin	2+	(−)
Red blood cell	5–10	0–2
White blood cell	0–2	0–2

## Discussion

The most common clinical presentations of SAM include abdominal pain and bleeding. The haemoperitoneum in our case is related to hepatocellular cell carcinoma with rupture; however, the tarry stool episode with unremarkable findings from panendoscopy of the upper gastrointestinal tract could suggest the underlying SAM [9–12]. Radiographical indications of SAM range from arterial dissections, fusiform aneurysms, arterial wall thickening, arterial stenosis and arterial occlusion [13, 14].

The differentiated diagnosis may include infection (e.g. mycotic aneurysm and endocarditis) and connective tissue diseases (e.g. polyarteritis nodosa, Kawasaki disease, primary granulomatous central nervous system vasculitis, Wegener granulomatosis, Churg-Strauss syndrome, microscopic polyangiitis, Henoch-Schönlein syndrome, systemic lupus erythematosus, rheumatoid vasculitis, fibromuscular dysplasia (FMD) and Behcet syndrome) [8, 15–18]. Of these, FMD mimics SAM due to its similar pathological appearance and angiographic findings and it has been deemed to be a precursor lesion of SAM—although predominantly in young females [8, 14].

Although angiographical patterns are suggestive of SAM, histopathology remains the ‘gold standard’ for definitive diagnosis. This is especially important in the case of *polyarteritis nodosa*, which can have an angiographical appearance identical to SAM. The treatment of SAM is composed of vascular embolization or surgical resection [2, 19]. The discrimination of SAM from systemic inflammatory vasculitides is particularly important since corticosteroids—and other immunosuppressive agents that are crucial in the treatment of the inflammatory vasculitides—have no proven benefits in SAM. However, rare case reports have correlated with malignancy and segmental arterial mediolysis [20, 21]. To the best of our knowledge, this is the first case report of the incidental finding of a ruptured hepatocelluar carcinoma accompanied with segmental arterial mediolysis.

Segmental arterial mediolysis is an acute, limited disease. In patients with malignancy-related intra-abdominal bleeding, it is difficult to locate the origin of bleeding—as in our case with a ruptured hepatocellular carcinoma. Conventional angiography is more sensitive than CT or magnetic resonance angiography, and should be used if the more commonly-used methods fail to reveal a diagnosis. If a diagnosis of SAM is suspected a multi-disciplinary approach—involving consultation with interventional radiology and vascular or general surgery—should be promptly pursued.

**Conflict of interest:** none declared.
